# The relationships between age, sex, and cerebrovascular reactivity to hypercapnia using traditional and kinetic-based analyses in healthy adults

**DOI:** 10.1152/ajpheart.00300.2022

**Published:** 2022-09-02

**Authors:** Jodie L. Koep, Bert Bond, Alan R. Barker, Stefanie L. Ruediger, Faith K. Pizzey, Jeff S. Coombes, Tom G. Bailey

**Affiliations:** ^1^Physiology and Ultrasound Laboratory in Science and Exercise, Centre for Research on Exercise, Physical Activity and Health, School of Human Movement and Nutrition Sciences, The University of Queensland, Brisbane, Queensland, Australia; ^2^Children’s Health and Exercise Research Centre, Sport and Health Sciences, College of Life and Environmental Sciences, University of Exeter, Exeter, United Kingdom; ^3^Exeter Head Impacts, Brain Injury and Trauma (ExHIBIT) Research Group, University of Exeter, Exeter, United Kingdom; ^4^School of Nursing, Midwifery and Social Work, The University of Queensland, Brisbane, Queensland, Australia

**Keywords:** aging, carbon dioxide, cerebral blood flow, internal carotid artery, life span

## Abstract

The effect of age and sex on intracranial and extracranial cerebrovascular function is poorly understood. We investigated the relationships between age, sex, and cerebrovascular reactivity (CVR) to hypercapnia in 73 healthy adults (18–80 yr, *n* = 39 female). CVR to hypercapnia was assessed in the middle cerebral artery (MCA) using transcranial Doppler ultrasound and at the internal carotid artery (ICA) using duplex ultrasound. MCA CVR was characterized by peak MCA velocity (MCAv) response per mmHg increase in end-tidal CO_2_ and by using a monoexponential model to characterize the kinetics (time constant) of the MCAv response. ICA reactivity was assessed as the relative peak increase in artery diameter. Hierarchical multiple regression determined the relationships between age, sex, and the age-by-sex interaction on all baseline and CVR outcomes. There was no relationship between ICA reactivity (%) with age (*P* = 0.07), sex (*P* = 0.56), or a moderator effect of sex on the age effect (*P* = 0.24). MCAv CVR showed no relationship with age (*P* = 0.59), sex (*P* = 0.09), or an age-by-sex moderator effect (*P* = 0.90). We observed a positive relationship of MCAv CVR time constant with age (*P* = 0.013), such that the speed of the MCA response was slower with advancing age. The present study provides comprehensive data on age- and sex-specific relationships with intracranial and extracranial cerebrovascular responses to hypercapnia. Despite similar MCAv CVR and ICA reactivity between sexes, kinetic responses of the MCA revealed a slower rate of adjustment with advancing age.

**NEW & NOTEWORTHY** We observed similar MCA CVR and ICA reactivity in males and females. However, kinetic responses of the MCA to hypercapnia suggest that advancing age slows down the rate at which MCA velocity increases in response to hypercapnia. These data indicate distinct regulatory differences, and an impaired vasomotor control of the cerebrovasculature with advancing age, not detected by traditional methods.

## INTRODUCTION

Cerebral blood flow (CBF) responsiveness to hypercapnia, termed cerebrovascular reactivity (CVR), is vital in stabilizing pH levels and maintaining delivery of oxygen and nutrients to the brain (1). Previous research has evidenced the clinical importance of this outcome, with a lower CVR later in life (60+ yr of age) associated with increased risk of age-associated disease including Alzheimer’s disease, cognitive decline (2), and all-cause mortality (3). Thus, it is important to understand the physiological changes in hypercapnia-induced CVR associated with advancing age.

Despite numerous studies investigating the effect of advancing age on CVR of the anterior circulation, conclusions remain unclear (4). Some studies demonstrate CVR to decline in older compared with young adults (5–8); however, other studies show CVR to remain unchanged in older adults (9–12) or even increase (13, 14). To date, research has largely ignored comparisons with middle-aged groups (∼40–60 yr), where regulatory, functional, and structural alterations to the vasculature may manifest, and the rate of decline in CVR might be greatest (5, 15). These disparate findings between studies may be underpinned by several factors including the method of assessment, nature of hypercapnia administration, or differences in the sample population, such as age, sex, and physical fitness, which may all independently influence CVR (16–19).

Burley et al. (16) showed that when they used transcranial Doppler (TCD) ultrasound, older adults had significantly greater CVR compared with younger adults. However, with blood oxygen level-dependent magnetic resonance imaging (BOLD-MRI) measures, no differences between groups were observed. Most previous studies used TCD and were limited by the reliance on velocity measures of a single intracranial vessel [middle cerebral artery (MCA)]. Previous findings using extracranial internal carotid artery (ICA) measures have shown a decreased ICA reactivity in older (68 ± 1 yr) compared with young (23 ± 11 yr) adults (20). However, given the small sample size of 20 participants, sex differences and the sex-dependent effect of age have yet to be adequately addressed.

Few studies investigate the effect of sex on CVR in aging adults (13, 14, 20). This is a vital consideration given the potential effects of estrogen and evidence that the effects of aging on the vasculature are sex-dependent (21). Carter et al. (22) demonstrated that in young females, MCAv CVR was greater compared with males; however, this sex difference was not evident in the ICA. This is consistent across some (18, 23), but not all (24, 25), studies, possibly due to the different methodologies and the use of TCD measures alone, as TCD relies on the assumption that the MCA diameter does not change (26). This, however, may not hold true during hypercapnia (27), with the magnitude of changes in dilation potentially influenced by age (6, 28) and sex (24). In contrast, Miller et al. (6), using MRI, demonstrated that decreases in intracranial artery responses to hypercapnia were evident with advancing age in males, but not females. This highlights the need to study the effects in males and females separately as not to confound interpretations on the effect of advancing age.

Recent research has highlighted the importance of investigating dynamic kinetic-based analyses on cerebrovascular regulation, in addition to traditional amplitude-based inferences (29). This can be achieved using a monoexponential model, where the time delay, time constant, and mean response time can provide additional information on the speed of the response (29, 30). These outcomes have been shown to be indicative of regulatory responses during exercise stressors (31, 32) but have yet to be applied to examine the effect of age and sex on cerebrovascular responses to hypercapnia.

The first aim of this study is to determine intracranial and extracranial CVR to hypercapnia across the healthy adult life span in males and females, exploring traditional CVR. The second aim is to investigate if any alterations in CVR with age are sex-dependent. We hypothesized that *1*) cerebrovascular reactivity in the intra- and extracranial vessels would show a negative relationship with age and *2*) the rate of decline would be sex-dependent, with higher CVR in both the MCA and ICA in younger females and a greater rate of decline with advancing age compared with males.

## METHODS

### Ethical Approval

All experimental procedures and protocols were approved by the University of Queensland Ethics Committee (No. 2019001863), and the study conformed to the standards set by the Declaration of Helsinki. Written, informed consent was obtained before participation in the study.

### Participants

Participant recruitment was based on an a priori power calculation to detect differences between age groups for ICA dilation (%) in response to hypercapnia, set for large effect size (*F* = 0.4), power (0.8), and α (0.05) (20) (G* Power 3.1 Kiel, Germany). This resulted in a target recruitment of 20 participants per age group (young = 18–39 yr; middle age = 40–64 yr; and older = 40–64 yr). Assuming a 20% data loss due to image capture/analysis problems, we set a target of ∼70–75 participants. Seventy-three adults between the ages of 18–80 yr volunteered to take part in this study.

Exclusion criteria included diagnosed arterial hypertension, smoking, any known cardiometabolic or respiratory diseases, the use of any prescribed medications known to influence cardiovascular function (e.g., statins, thyroid medication), and a body mass index (BMI) > 35 kg/m^2^. Any premenopausal females with an irregular menstrual cycle or the use of progesterone-only contraceptive pill were excluded from the study. In addition, naturally menstruating premenopausal females (*n* = 8) were tested in the follicular phase (1–14 days) to allow better comparisons between sexes (33). Females on the combined contraceptive pill (*n* = 10) were tested during the inactive pill phase (*days 1–7*). Postmenopausal females on hormone replacement therapy were excluded from the study.

### Study Design

Following baseline screening, participants completed one visit to the laboratory. They were required to fast for a minimum of 3 h and refrain from nitrate-rich foods for 12 h before testing. In addition, participants were required to avoid vigorous physical activity, caffeine, and alcohol consumption for 24 h before testing. Body mass and stature were measured according to standard procedures to the nearest 0.1 kg and 0.1 cm, respectively. BMI classifications were used to determine the weight status of participants (34). Physical activity levels were assessed via the Active Australia survey (35) and reported as METmin/wk, which accounts for time spent in different intensities of aerobic activity (36). This survey has been validated against pedometer and accelerometery data in healthy middle-aged adults (*R* = 0.52) (37) and compares favorably to other self-reported physical activity surveys (ICC = 0.64) (38). Female participants self-reported menopausal status via a questionnaire and were categorized into either premenopausal (regular periods), perimenopausal (irregular cycles), early postmenopausal [1–3 yr following last menstrual period (LMP)], or postmenopausal (6+ yr LMP) (39). Following initial screening and questionnaires, participants were required to rest in a darkened temperature-controlled laboratory (∼23°C) for 15 min in the supine position before instrumentation and the commencement of the protocol.

### Experimental Measures

The CVR protocol was conducted in the supine position in line with recommendations (40) and to replicate existing studies, given the potential effects of body position on CBF outcomes (41). It consisted of a 2-min baseline breathing ambient room air, followed by 5 min of hypercapnia. During hypercapnia, 5% CO_2_ was administered with 21% O_2_ (balanced nitrogen). This replicates the protocol from other laboratories which have investigated the effects of hypercapnia on advancing age (8) and within the normal vasodilatory stimuli ranges of 5–7% CO_2_ (42). A three-way valve (Hans Rudolph, Shawne, KS) allowed inspiratory gases to be switched from ambient air to the 5% CO_2_ mixture (using a 170-L Douglas bag, Hans Rudolph). Participants were instructed to breathe normally during hypercapnia and the baseline periods. Cardiorespiratory parameters were simultaneously determined throughout the protocol, as described in *Cardiorespiratory Measures*. Hypercapnia was chosen as the stimulus because of its sensitivity to disease risk (42) and the availability of reliability data on this outcome (30).

### Cardiorespiratory Measures

During the protocol, beat-by-beat blood pressure was continuously measured by finger volume-clamp method (Finapress, NOVA, The Netherlands). Participants wore a snorkel mouthpiece and nose clip (Hans Rudolph) to measure end-tidal carbon dioxide ($\text{P}{\text{ET}}_{\text{C}{\text{O}}_{2}}$) and end-tidal oxygen concentrations ($\text{P}{\text{ET}}_{{\text{O}}_{2}}$) using a gas analyzer (ADInstruments, ML206, Colorado Springs, CO), which was calibrated before each participant via known concentrations of O_2_ and CO_2_. V̇_E_ was measured using a spirometer (ADInstruments), calibrated with a 3-L syringe. Heart rate was assessed using a three-lead ECG (Finapress, NOVA, The Netherlands). All data were sampled continuously (PowerLab; Model No. 8/30, ADInstruments) and stored at 200 Hz using an analog-to-digital converter interfaced with a laptop computer (LabChart version 8, ADInstruments) for offline analysis.

### Cerebrovascular Measures

#### Intracranial.

A 2-MHz transcranial Doppler ultrasound probe (Spencer Technologies, ST3, Redmond, WA) was used to insonate the right MCA at an initial depth of ∼50 mm through the trans-temporal window using previously described guidelines (43). The Doppler signals were acquired, optimized, and secured using an adjustable headset (adult M600 bilateral head frame; Spencer Technologies, Redmond, WA). Beat-by-beat MCAv was calculated as the mean across each cardiac cycle and exported from LabChart as second-by-second data for analysis (Version 8, ADInstruments).

#### Extracranial.

Diameter and mean blood velocity were measured in the right ICA using a 12-MHz linear-array Doppler probe through a high-resolution ultrasound machine (Terason, 3300, U-smart, Burlington, MA). The ICA was identified, and the image and waveform were optimized in accordance with extracranial carotid artery guidelines (40). Doppler velocity assessments were obtained using pulse-wave mode with an insonation angle ≤60°. Following optimization of the longitudinal B-mode image of the arterial walls, images of the artery and associated velocity waveforms were simultaneously recorded during the baseline and hypercapnic periods.

### Data Processing

#### Steady-state response.

Baseline values were averaged over 120 s of supine rest. All data from LabChart were exported as 1-s averages into Excel (Microsoft, Seattle, WA). Analysis of ICA diameter and flow were performed using custom-designed, edge-detection, and wall-tracking software (BloodFlow Analysis, version 5.1). This approach is independent of investigator bias with automated wall tracking and has previously been comprehensively described and validated (44, 45). Analysis was performed blinded to the participant age and sex. From synchronized diameter and velocity data, blood flow (the product of lumen cross-sectional area and Doppler velocity), and shear rate (4 × mean blood velocity/vessel diameter) were calculated at 30 Hz (46). ICA data were then interpolated from 30 to 1 Hz and exported into an Excel spreadsheet. The LabChart and ICA vascular data were time aligned to the start of the hypercapnic protocol in LabChart and reexported to Excel for subsequent analysis using in-house, carotid shear-mediated dilation software. This automated software calculated indices of baseline (median value of the baseline), peak response following the onset of CO_2_, and the relative change (%) from baseline to peak, for all ICA variables (diameter, peak systolic velocity, mean blood flow, and shear rate).

Flow pulsatility index (PI) was calculated as the difference between diastolic and systolic MCAv divided by the mean MCAv (MCAv_systolic_ – MCAv_diastolic_/MCAv_mean_) (47). The PI response was obtained at baseline and the final 30 s of each minute of hypercapnia. Cerebrovascular resistance index (CVRi) was calculated as mean arterial pressure (MAP) divided by MCAv (mmHg/cm/s) at baseline and the last 30 s of hypercapnia to appropriately capture the hypercapnic response, given that CVRi decreases throughout the response. The responses to hypercapnia were obtained in the final 30 s of each minute, and differences from baseline to peak during hypercapnia were calculated for HR, MAP, ICA blood flow, MCAv, V̇e, $\text{P}{\text{ET}}_{\text{C}{\text{O}}_{2}}$, and $\text{P}{\text{ET}}_{{\text{O}}_{2}}$.

Calculation of CVR to hypercapnia was expressed as the absolute change from baseline MCAv per unit increase (mmHg) in $\text{P}{\text{ET}}_{\text{C}{\text{O}}_{2}}$. This response was quantified as the peak rolling 30-s average during hypercapnia, wherever this occurred (30). CVR was calculated in this way as the most reliable analysis method to address recent concerns on the variability of changes in MCAv during open-circuit breathing (30, 48).

#### Kinetic response.

**Figure 1.**
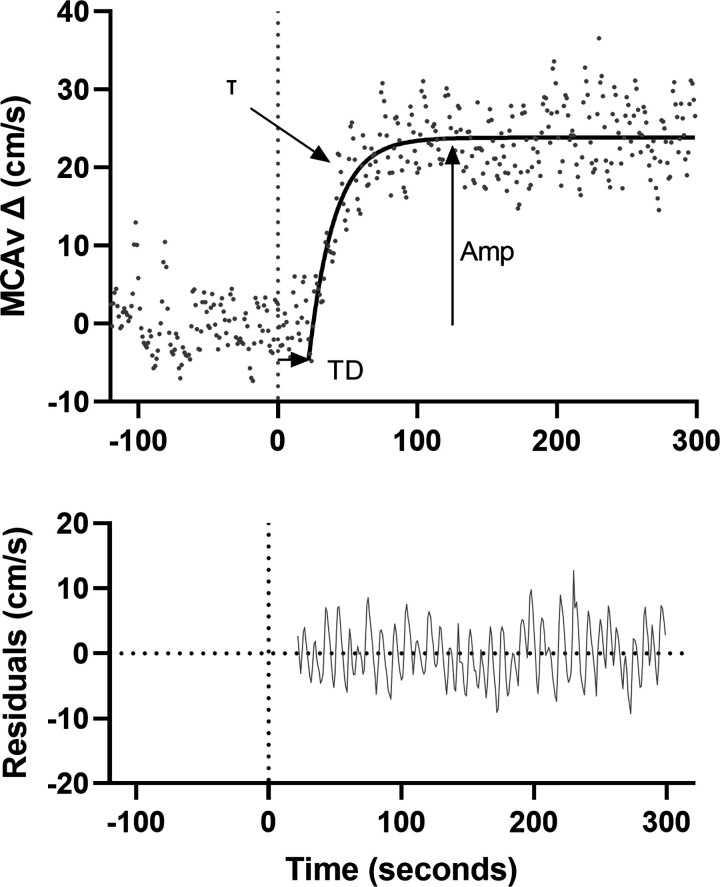
An example of MCAv trace for one participant at rest (120 s) and following onset of hypercapnia (*time 0*, denoted by dotted line). Residual plot is included below. Hypercapnic response characterized by a monoexponential model with a delay term shown by the solid black line. Time delay (TD) presents the time from the start of hypercapnia (0 s) to the onset of the exponential rise. Time constant (τ) presents the time taken to reach 63% of the response amplitude and it reflects the rate of increase in MCAv. Amplitude (Amp) presents the change from baseline to the peak of the exponential increase in MCAv. MCAv, middle cerebral artery velocity.

Data were baseline corrected for the 120 s preceding hypercapnia and analyzed using a monoexponential model with time delay using GraphPad Prism (Fig. 1) (GraphPad Software, San Diego, CA) as follows: MCAv(*t*) = ΔMCAvA[1 – *e*^–(^*^t^*^ − TD/^τ^)^], where MCAv(*t*) is the MCAv at a given time (*t*), ΔMCAvA is the amplitude change of MCAv from baseline to its asymptote, TD is the time delay, and τ is the time constant, in accordance with kinetic modeling in previous work (29, 30). Mean response time (MRT) was calculated as the sum of the model derived τ and the TD. The model was fitted from the start of the exponential rise until a deviation from a subjective visual steady state was observed. All models were then checked by two independent researchers for consistency, and any disagreements were discussed until a consensus was reached. Acceptability of appropriate fit was determined as the goodness of fit *R*^2^ > 0.50 and normality of residuals. The precision of the derived τ was quantified using 95% confidence intervals.

#### Internal carotid artery dilation.

All ICA data were passed through a two-stage filtering process; a median filter (with a rank of 7) was applied to the parameter data, followed by passage through a Savitzky–Golay finite impulse-response smoothing filter with a window size of 13 data points and a polynomial order of 1 (22). These filters removed high-frequency noise to reveal the underlying lower frequency physiological response profiles. All subsequent analyses were performed using this graphed, filtered data of variables including ICA shear rate, diameter, mean blood flow, peak blood flow, mean velocity, and peak systolic velocity. The following variables were automatically detected and calculated by the software: *1*) baseline, the median value of the 2-min baseline period preceding hypercapnia; *2*) peak response, the autodetected maximum value of the filtered data identified after the onset of CO_2_; and *3*) relative change, change from baseline to peak, calculated as [(peak − baseline)/baseline] ×100. The total and initial stimulus for subsequent dilation were quantified as the shear rate (SR) area under the curve (AUC) from the time of CO_2_ onset to the time of peak diameter (SR_AUC_) and to the first 50 s after CO_2_ onset (SR_AUC-initial_). SR_AUC-initial_ was chosen as an attempt to account for the initial shear stress stimulus, driving the potential changes in diameter. SR_AUC_ and SR_AUC-50s_ were calculated using the trapezoid rule (GraphPad, Prism, version 9). In addition, a thresholding algorithm was applied to each data array (e.g., ICA shear, ICA diameter), which identified threshold points. These thresholds were defined as the point at which each variable began to systematically increase, above the baseline level, after the application of the CO_2_ stimulus. The threshold point was calculated as follows: threshold point = (maximum value – minimum value) × %variation factor) + baseline median value (20, 22). This variation factor was chosen to ensure that the variable had increased to a point that represented a definitive deviation from baseline, which also exceeded fluctuations associated with cardiac and respiratory cycles. Once the software had automatically detected the threshold points, they were depicted on the raw data array and visually inspected to ensure they met the following criteria: *1*) the algorithm-detected threshold point occurred before the peak value and *2*) the variable did not decrease below the algorithm-detected threshold point before the peak value occurring (22). In cases where they did not meet the criteria (∼22%), the threshold points were manually adjusted independently to a point where it was deemed there was a clear deviation from baseline values that met the above criteria. This was then checked by two independent researchers.

ICA dilation (%) was allometrically scaled to account for differences in baseline ICA diameter as previously described (49, 50).

### Statistical Analyses

Statistical analyses were conducted using SPSS (version 25; IBM, Armonk, NY). All data are presented as means ± SD. Differences in participant characteristics were explored using a two-way analysis of variance (ANOVA) with sex (male, female) and age (young, middle age, older) as the independent variables. For *aim 1*, hierarchical multiple regression was used to determine the relationships between age (yr; *model 1*) and sex (*model 2*) with all baseline and hypercapnic variables of interest. For *aim 2*, an interaction term of age × sex (*model 3*) was added to address whether sex moderated the effects of age by assessing the differences in regression slope coefficients between males and females on all variables of interest. The outputs for *model 1* and 2 included the slope coefficient (unstandardized β) and the explained variance of the full model (*R*^2^) and the significance of the relationship (*P* value). For *model 3*, the output included slope coefficients (unstandardized β) of the interaction terms for males by age and for females by age. The *P* value describes whether there was a significant difference in slope coefficients between males and females with advancing age (significant interaction), and the *R*^2^ denotes the degree of explained variance of the entire regression model with the interaction term included.

To adjust for any variance explained by body mass and physical activity levels, BMI (kg/m^2^) and self-reported physical activity (METmin/wk) were added to the model. Finally, MAP was added to the model to adjust for any variance on cerebrovascular and ICA outcomes explained by changes in perfusion pressure. In instances where these factors (BMI, physical activity, and MAP) significantly explained any variance in the overall model response, the results are presented for the full model and whether the addition of this variable altered the effects of age, sex, and the age-by-sex interaction term. A simple linear regression was run to investigate the influence of menopause on variables of interest in a female-only model. The model investigated the relationship between early postmenopause (1–3 yr LMP) and late postmenopause (6+ yr LMP) to a reference group of premenopausal females on variables of interest. All data were normally distributed as assessed by visual inspection of Q-Q plots and homoscedasticity of the studentized residuals plotted against the predicted values. Linearity was established by visual inspection of a scatterplot. There was no evidence of multicollinearity as evidenced by no tolerance values less than 0.24. Although some data points were identified as above two to three standard deviations from the mean, none was deemed implausible and removed. Statistical significance was accepted at an α of *P* < 0.05.

## RESULTS

Participants were recruited into young (*n* = 25, 12 female, age = 27.0 ± 2.6 yr, range = 22–32 yr), middle-aged (*n* = 30, 17 female, age = 52.9 ± 7.5 yr, range = 35–63 yr), and older (*n* = 18, 10 female, age = 69.8 ± 3.5 yr, range = 65–77 yr) groups. Participant characteristics of the cohort can be seen in Table 1. Intracranial kinetic analyses are presented for 71 adults (38 females), because of unacceptable model fit in two individuals. Extracranial analyses are included for 58 individuals (31 females). Reasons for data loss of ICA analyses were the inability to obtain a sufficiently clear ultrasound image in 15 individuals.

**Table 1.** Participant characteristics and baseline cerebrovascular parameters

	Total		Young		Middle		Older	
Characteristics	Males	Females	Males	Females	Males	Females	Males	Females
*n*	34	39	13	12	13	17	8	10
Age, yr	46.9 ± 18.5	50.6 ± 16.5	26.8 ± 2.9^a,b^	27.2 ± 2.3^a,b^	50.3 ± 8.4^a,c^	54.7 ± 6.5^a,c^	70.9 ± 3.3^b,c^	70.0 ± 3.5^b,c^
Stature, cm	175.7 ± 9.4*	171.5 ± 7.9	179.4 ± 6.7	172.0 ± 7.9	170.2 ± 9.9	174.4 ± 7.5	178.4 ± 8.8	165.7 ± 5.5
Weight, kg	77.0 ± 13.7	73.7 ± 13.5	77.7 ± 13.0	79.0 ± 17.7	70.3 ± 11.1	72.1 ± 11.5	85.8 ± 14.0	70.2 ± 10.0
BMI, kg·m^2^	21.8 ± 3.2	21.4 ± 3.3	21.6 ± 3.4	22.9 ± 4.3	20.6 ± 2.5	20.6 ± 2.7	24.0 ± 3.1	21.2 ± 2.9
Normal weight, %^Ω^	75	74	62	67	85	76	75	80
PA, METmin/wk	2,358 ± 1,590	2,029 ± 1,071	2,172 ± 829	2,309 ± 1,159	1,867 ± 1,076	1,944 ± 992	3,373 ± 2,579	1,859 ± 1,151
MAP, mmHg	96 ± 19	103 ± 17	87 ± 26^a,b^	93 ± 11^a,b^	97 ± 10^a,c^	103 ± 15^a,c^	108 ± 7^b,c^	111 ± 13^b,c^
*Intracranial cerebrovascular parameters (n = 73, females = 39)*								
MCAv, cm/s	63.7 ± 14.0*	72.7 ± 15.9	70.8 ± 14.0^b^	76.9 ± 10.9^b^	62.8 ± 12.2^c^	74.8 ± 15.0^c^	53.7 ± 11.3^b,c^	64.2 ± 20.2^b,c^
CVCi, cm/s/mmHg	0.65 ± 0.19*	0.74 ± 0.21	0.77 ± 0.18^a,b^	0.88 ± 0.16^a,b^	0.64 ± 0.15^a,c^	0.73 ± 0.16^a,c^	0.48 ± 0.14^b,c^	0.58 ± 0.23^b,c^
CVRi, mmHg/cm/s	1.6 ± 0.5	1.5 ± 0.5	1.3 ± 0.4^a,b^	1.2 ± 0.2^a,b^	1.6 ± 0.4^a,c^	1.4 ± 0.3^a,c^	2.1 ± 0.5^b,c^	2.0 ± 0.6^b,c^
PI, AU	0.76 ± 0.16†	0.74 ± 0.12	0.82 ± 0.22	0.67 ± 0.10	0.69 ± 0.10^c^	0.72 ± 0.10^c^	0.78 ± 0.08^c^	0.85 ± 0.11^c^
$\text{P}{\text{ET}}_{\text{C}{\text{O}}_{2}}$, mmHg	41.7 ± 6.2	41.4 ± 8.8	44.6 ± 5.4	42.4 ± 4.6	42.9 ± 4.4	41.2 ± 4.6	36.7 ± 6.0	40.5 ± 4.9
V̇e, L/min	11.7 ± 4.2	10.8 ± 4.2	11.9 ± 4.1	11.4 ± 4.3	11.6 ± 5.2	11.4 ± 4.8	11.3 ± 2.7	9.2 ± 2.8
*Extracranial internal carotid artery parameters (n = 58, females = 31)*								
ICA diameter, cm	0.61 ± 0.11*	0.52 ± 0.08	0.60 ± 0.10	0.49 ± 0.05	0.56 ± 0.03	0.54 ± 0.08	0.68 ± 0.16	0.53 ± 0.10
Peak systolic velocity, cm/s	30.9 ± 7.7	36.2 ± 12.5	29.8 ± 7.5	34.4 ± 9.9	31.4 ± 8.1	35.2 ± 10.6	31.9 ± 8.4	40.2 ± 18.5
Mean blood flow, mL/min	470.8 ± 235.8	417.1 ± 270.0	476.4 ± 267.2	266.4 ± 98.5	395.3 ± 221.0	535.7 ± 388.9	566.3 ± 200.8	412.3 ± 199.5
Shear rate, s^−1^	213.1 ± 69.2*	268.2 ± 86.5	207.7 ± 57.4	287.7 ± 104.0	222.4 ± 50.9	256.4 ± 80.6	206.9 ± 106.9	263.8 ± 78.0

Values are means ± SD. Data were compared using a two-way ANOVA with main effects of age (males and females) and sex (young, middle, and older). *Significant main effects of sex. When main effect of age is present, post hoc pairwise comparisons reveal where significant differences lie: ^a^young vs. middle, ^b^young vs. older, ^c^middle vs. older. †Significant age-by-sex interaction effect. ^Ω^According to BMI classifications [Weir and Jan (34)], proportion of participants classified as normal weight are presented as a percentage. BMI, body mass index; CVCi, cerebrovascular conductance index; CVRi, cerebrovascular resistance index; MAP, mean arterial pressure; MCAv, Middle cerebral artery velocity; PA, physical activity; $\text{P}{\text{ET}}_{\text{C}{\text{O}}_{2}}$, end-tidal carbon dioxide; PI, pulsatility index; V̇e, minute ventilation.

The main effects of age and sex on participant characteristics are highlighted in Table 1. A significant age-by-sex interaction was present for PI (*P* = 0.006). Post hoc pairwise comparisons revealed significant differences between young males compared with females (*P* = 0.004). In male participants, significant differences between young compared with middle-aged adults were present (*P* = 0.008). In female participants, significant differences between older adults compared with young (*P* = 0.001) and middle-aged adults were observed (*P* = 0.01).

### Baseline Responses

The relationships between age and baseline cerebrovascular and cardiorespiratory variables of interest can be seen in Table 2. These data are presented for age (*model 1*), age and sex (*model 2*), and the moderator effect of sex on the relationship between age and variables of interest (*model 3*).

**Table 2.** Baseline cardiovascular and cerebrovascular variables

	*Model 1*: Age			*Model 2*: Age and Sex					*Model 3*: Interaction (Age × Sex)			
				Age		Sex						
	*P* value	*R* ^2^	β	*P* value	β	*P* value	β	Combined *R*^2^	*P* value	*R* ^2^	β Males	β Females
*Intracranial cerebrovascular parameters (n = 73, females = 39)*												
MCAv, cm/s	**0.005**	0.11	−0.29 ± 0.10	**0.001**	−0.33 ± 0.09	**0.003**	10.2 ± 3.3	0.21	0.41	0.22	−0.41 ± 0.13	−0.25 ± 0.14
CVCi, cm/s/mmHg	**<0.001**	0.33	−0.006 ± 0.001	**<0.001**	−0.007 ± 0.001	**0.006**	0.11 ± 0.04	0.37	0.80	0.37	−0.007 ± 0.002	−0.006 ± 0.002
CVRi, mmHg/cm/s	**<0.001**	0.30	0.016 ± 0.003	**<0.001**	0.016 ± 0.003	0.09	0.17 ± 0.10	0.33	0.49	0.34	0.014 ± 0.004	0.018 ± 0.004
PI, AU	0.29	0.016	0.001 ± 0.001	0.25	0.001 ± 0.001	0.42	−0.027 ± 0.033	0.025	**0.002**	0.14	−0.001 ± 0.001	0.004 ± 0.001
*Cardiovascular parameters (n = 73, females = 39)*												
MAP, mmHg	**<0.001**	0.25	0.49 ± 0.10	**<0.001**	0.47 ± 0.10	0.15	5.20 ± 3.50	0.27	0.92	0.27	0.48 ± 0.14	0.46 ± 0.15
$\text{P}{\text{ET}}_{\text{C}{\text{O}}_{2}}$, mmHg	0.06	0.048	−0.09 ± 0.05	0.07	−0.10 ± 0.05	0.93	−0.15 ± 1.7	0.048	0.16	0.075	−0.16 ± 0.07	−0.02 ± 0.07
V̇e, L/min	0.28	0.02	−0.031 ± 0.03	0.31	−0.029 ± 0.03	0.57	−0.57 ± 0.9	0.02	0.45	0.03	−0.009 ± 0.04	−0.05 ± 0.04
*Extracranial internal carotid artery parameters (n = 58, females = 31)*												
ICA diameter, cm	0.09	0.05	0.001 ± 0.001	0.08	0.002 ± 0.001	**0.001**	−0.09 ± 0.02	0.23	0.73	0.23	0.002 ± 0.001	0.001 ± 0.001
Peak systolic velocity, cm/s	0.31	0.02	0.08 ± 0.08	0.44	0.06 ± 0.06	0.07	5.4 ± 2.8	0.08	0.70	0.08	0.094 ± 0.12	0.03 ± 0.12
Mean blood flow, mL/min	0.14	0.04	2.9 ± 1.9	0.12	3.0 ± 1.9	0.38	−60.5 ± 67.9	0.06	0.24	0.08	0.91 ± 2.7	5.5 ± 2.8
Shear rate, s^−1^	0.48	0.009	−0.47 ± 0.65	0.34	−0.60 ± 0.62	**0.007**	59.2 ± 21.3	0.14	0.49	0.14	−0.17 ± 0.8	−1.0 ± 0.9

Values are means ± SD. Boldface indicates significant relationship (*P* < 0.05). *Model 1* presents the relationship between age and the indicated variable. *Model 2* presents the addition of sex to the model and the relationship between age and sex with the indicated variable. *Model 3* indicates if sex moderates the relationship between age and the indicated variable, with individual unstandardized β Coefficients shown for males and females. In *models 1–3*, *R*^2^ value reflects the full model. In *models 1* and *3*, β represents the unstandardized β coefficient representing the change in variable units for every year increase in age. In *model 2*, the β coefficient provides the difference in females vs. males in the variable units. CVCi, cerebrovascular conductance index; CVRi; cerebrovascular resistance index; ICA, internal carotid artery; MAP, mean arterial pressure; MCAv, middle cerebral artery velocity; $\text{P}{\text{ET}}_{\text{C}{\text{O}}_{2}}$, end-tidal carbon dioxide; PI, pulsatility index; V̇e, minute ventilation.

**Figure 2.**
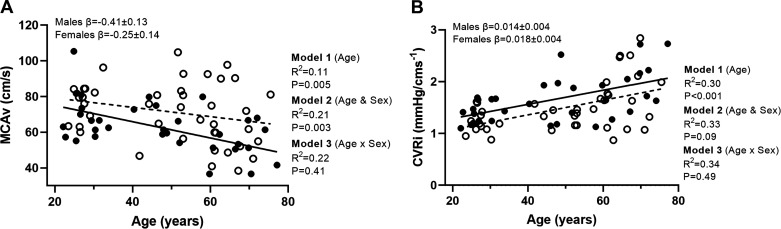
*A* and *B*: linear regression analysis demonstrating the relationships between age and baseline middle cerebral artery velocity (MCAv; *A*) and cerebrovascular resistance index (CVRi; *B*; *n =* 73, females = 39). Solid line represents the regression fit for males, and dotted line represents the regression fit for female participants. Hierarchical regression models (*P* value and *R*^2^) are presented for the relationship with age (*model 1*), the relationship with sex (*model 2*), and the moderator relationship of the sex-dependent relationship with age (*model 3*). Slope coefficients (β) of the relationship with age are presented for males and females separately (*model 3*). MCAv, middle cerebral artery velocity.

There was a negative relationship between MCAv and age (*model 1*). With the addition of sex to the model (*model 2*), this showed a relationship between MCAv and sex, explaining 21% of variance (*P* = 0.003) and MCAv higher in females (β = 10.2 ± 3.3 cm/s; Fig. 2*A*, Table 2). There was no age-by-sex interaction (*model 3*) for baseline MCAv (*P* = 0.41). There was a positive relationship between CVRi and age (*P* < 0.001; *R*^2^ = 0.30; Fig. 2*B*); with the addition of sex in *model 2*, this did not lead to an increase in explained variance for CVRi (*P* = 0.09). Sex did not moderate the effect of age on baseline CVRi (*P* = 0.49).

Baseline ICA diameter showed no relationship with age (*P* = 0.09), but there was an effect of sex, with the ICA diameter greater in males and explaining 23% of variance (*P* < 0.001). Sex did not moderate the effect of age on baseline ICA diameter such that the slope coefficients were not different in males and females with age (*P* = 0.73).

Baseline ICA shear rate, peak systolic velocity, and mean blood flow all showed no relationship with age (*P* ≥ 0.14). With the addition of sex to the model, there was a relationship for shear rate (*P* = 0.007). All other ICA variables (peak systolic velocity and mean blood flow) showed no relationship with the addition of sex to the model (*P* ≥ 0.07). Sex did not moderate the effects of age on baseline shear rate, peak systolic velocity, and mean blood flow (*P* ≥ 0.24; Table 2).

For baseline $\text{P}{\text{ET}}_{\text{C}{\text{O}}_{2}}$, with the addition of physical activity (weekly METmin) to the model, this led to an increase in explained variance of the full model (*R*^2^ = 16, *P* = 0.04; β = −0.005 ± 0.002). The addition of physical activity to the model did not significantly influence the effects of age (*R*^2^ = 0.052; *P* = 0.05), sex (*R*^2^ = 0.052; *P* = 0.97), or the interaction of age and sex on baseline $\text{P}{\text{ET}}_{\text{C}{\text{O}}_{2}}$ (*R*^2^ = 0.095; *P* = 0.17). For all other baseline cardiovascular and cerebrovascular outcomes, the addition of physical activity and BMI did not lead to an increase in explained variance and therefore was not included in the model (*R*^2^ ≤ 0.41; *P* ≥ 0.057). With the addition of MAP to the model, this led to an increase in explained variance for baseline MCAv (*R*^2^ = 0.28; *P* = 0.03; β = 0.25 ± 0.11). The addition of MAP to the model did not influence any of the relationships between baseline MCAv and age (*R*^2^ = 0.11; *P* = 0.005), sex (*R*^2^ = 0.21; *P* = 0.003), or the interaction of age and sex (*R*^2^ = 0.22; *P* = 0.41). There was no relationship between MAP and any ICA baseline responses (*R*^2^ ≤ 0.02; *P* ≥ 0.26).

### Peak Hypercapnia Responses

The relationship between age and sex and the peak hypercapnic cerebrovascular and cardiorespiratory variables of interest can be seen in Table 3. These data are presented for age (*model 1*), age and sex (*model 2*), and the moderator effect of sex on the relationship between age and variables of interest (*model 3*). Data for cardiovascular and cerebrovascular responses to hypercapnia are presented in Supplemental Table S1; see https://doi.org/10.6084/m9.figshare.20422593).

**Table 3.** Peak cardiovascular and cerebrovascular responses to hypercapnia

	*Model 1*: Age			*Model 2*: Age and Sex					*Model 3*: Interaction (Age × Sex)			
				Age		Sex						
	*P* value	*R* ^2^	β	*P* value	β	*P* value	β	Combined *R*^2^	*P* value	*R* ^2^	β Males	β Females
*Intracranial cerebrovascular parameters*												
MCAv, cm/s	**0.03**	0.07	−0.30 ± 0.13	**0.007**	−0.35 ± 0.13	**0.002**	14.0 ± 4.4	0.18	0.28	0.20	−0.49 ± 0.18	−0.21 ± 0.18
CVR, cm^−1^/mmHg	0.59	0.004	−0.003 ± 0.006	0.45	−0.004 ± 0.006	0.09	0.34 ± 0.20	0.05	0.90	0.05	−0.005 ± 0.008	−0.004 ± 0.008
PI, AU	0.45	0.008	0.003 ± 0.004	0.47	0.003 ± 0.004	0.74	0.046 ± 0.14	0.01	0.72	0.01	0.001 ± 0.005	0.004 ± 0.006
CVCi, cm/s/mmHg	**<0.001**	0.26	−0.007 ± 0.001	**<0.001**	−0.007 ± 0.001	**0.01**	0.11 ± 0.05	0.32	0.83	0.32	−0.007 ± 0.002	−0.007 ± 0.002
CVRi, mmHg/cm/s	**<0.001**	0.24	0.012 ± 0.003	**<0.001**	0.013 ± 0.003	0.07	0.16 ± 0.09	0.27	0.43	0.28	0.015 ± 0.004	0.011 ± 0.004
*Cardiovascular parameters*												
MAP, mmHg	**<0.001**	0.31	0.48 ± 0.10	**<0.001**	0.47 ± 0.12	0.34	3.24 ± 3.32	0.32	0.30	0.33	0.36 ± 0.14	0.57 ± 0.13
$\text{P}{\text{ET}}_{\text{C}{\text{O}}_{2}}$, mmHg	0.32	0.01	−0.14 ± 0.13	0.28	−0.15 ± 0.14	0.43	−3.9 ± 4.8	0.02	0.77	0.02	−0.12 ± 0.20	−0.19 ± 0.19
V̇e, L/min	0.74	0.002	−0.017 ± 0.05	0.93	−0.005 ± 0.05	**0.04**	−3.8 ± 1.8	0.06	0.99	0.06	−0.005 ± 0.05	−0.004 ± 0.08
*Extracranial internal carotid artery parameters*												
ICA diameter dilation, %	0.07	0.06	0.043 ± 0.02	0.06	0.044 ± 0.02	0.56	−0.47 ± 0.79	0.07	0.24	0.09	0.018 ± 0.032	0.072 ± 0.033
Peak velocity, cm/s	0.09	0.05	0.21 ± 0.12	0.13	0.19 ± 0.12	0.14	6.43 ± 4.29	0.09	0.41	0.10	0.29 ± 0.17	0.09 ± 0.17
Peak velocity, Δ%	0.21	0.03	0.27 ± 0.21	0.18	0.27 ± 0.21	0.43	−6.0 ± 7.5	0.04	**0.02**	0.13	0.78 ± 0.29	−0.20 ± 0.29
Mean blood flow, mL/min	**0.02**	0.11	7.1 ± 2.8	**0.01**	7.4 ± 2.8	0.20	−128.7 ± 98.0	0.13	0.86	0.13	6.9 ± 3.9	8.0 ± 4.1
Mean blood flow, Δ%	**0.03**	0.08	0.50 ± 0.23	**0.03**	0.51 ± 0.23	0.56	−4.8 ± 8.2	0.09	0.10	0.13	0.87 ± 0.32	0.11 ± 0.34
Shear rate, s^−1^	0.97	0.00	−0.036 ± 1.02	0.80	−0.24 ± 0.96	**0.008**	91.82 ± 33.17	0.13	0.11	0.17	1.30 ± 1.33	−1.82 ± 1.35
Shear rate, Δ%	0.10	0.05	0.38 ± 0.23	0.10	0.39 ± 0.23	0.57	−4.5 ± 7.9	0.05	**0.01**	0.17	0.96 ± 0.31	−1.91 ± 0.31
SR_AUC_, s^−1^	0.17	0.03	−140.7 ± 101.5	0.14	−152.5 ± 101.7	0.25	4136.7 ± 3563.9	0.06	0.53	0.07	−90.37 ± 141.33	−220.80 ± 148.18
SR_AUC initial_, s^−1^	0.83	0.001	−2.74 ± 12.93	0.68	−5.29 ± 12.63	0.05	894.89 ± 442.92	0.07	0.07	0.13	16.34 ± 17.09	−29.07 ± 17.92

Values are means ± SD. Boldface indicates significant relationship (*P* < 0.05). *Model 1* presents the relationship between age and the indicated variable. *Model 2* presents the addition of sex to the model and the relationship between age and sex with the indicated variable. *Model 3* indicates if sex moderates the relationship between age and the indicated variable, with individual β coefficients shown for males and females. In *models 1–3*, *R*^2^ value reflects the full model. In *models 1* and *3*, β represents the unstandardized β coefficient representing the change in variable units for every year increase in age. In *model 2*, the Δ β coefficient provides the difference in females vs. males in the variable units. CVCi, cerebrovascular conductance index; CVR, cerebrovascular reactivity; CVRi; cerebrovascular resistance index; ICA, internal carotid artery; MAP, mean arterial pressure; MCAv, middle cerebral artery velocity; $\text{P}{\text{ET}}_{\text{C}{\text{O}}_{2}}$, end-tidal carbon dioxide; PI, pulsatility index; SR_AUC_, shear rate area under the curve; V̇e, minute ventilation.

There was a negative relationship between age and peak MCAv (*P* = 0.03; Table 3). With the addition of sex to the model, there was a significant relationship explaining an additional 11% of variance (*P* = 0.002) with peak MCAv greater in females. Sex did not moderate the effect of age on peak MCAv (*P* = 0.28). With the addition of MAP to the model, this led to an increase in explained variance for peak MCAv (*R*^2^ = 0.26; *P* = 0.02). The addition of MAP to the model did not alter the relationship with age (*R*^2^ = 0.07; *P* = 0.03), sex (*R*^2^ = 0.18; *P* = 0.002), and the interaction of age and sex (*R*^2^ = 0.20; *P* = 0.28).

**Figure 3.**
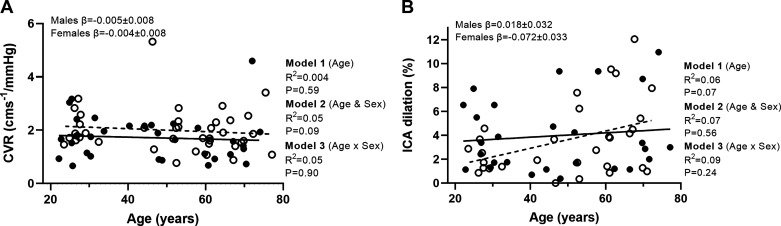
*A* and *B*: linear regression analysis demonstrating the relationships between age and CVR (*n =* 73, females = 39; *A*) and %ICA dilation and age (*n =* 58, females = 31; *B*). Solid line represents the regression fit for males, and dotted line represents the regression fit for female participants. Hierarchical regression models (*P* value and *R*^2^) are presented for the relationship with age (*model 1*), the relationship with sex (*model 2*), and the moderator relationship of the sex-dependent relationship with age (*model 3*). Slope coefficients (β) of the relationship with age are presented for males and females separately (*model 3*). CVR, cerebrovascular reactivity; ICA, internal carotid artery.

MCAv CVR (cm/mmHg) showed no relationship with age (*P* = 0.59), sex (*P* = 0.09), or any moderator effect (*P* = 0.90; Table 3, Fig. 3*A*). With the addition of physical activity (weekly METmin) to the model, this did not lead to an increase in explained variance (*R*^2^ = 0.05; *P* = 0.05) and did not influence the relationships with age (*P* = 0.84; *R*^2^ = 0.001), sex (*P* = 0.08; *R*^2^ = 0.05), or the moderator effect (*P* = 0.94; *R*^2^ = 0.05). With the addition of MAP to the model, an increase in explained variance for MCAv CVR was observed (*R*^2^ = 0.13; *P* = 0.01). This did not alter the relationships between MCAv CVR with age (*P* = 0.59; *R*^2^ = 0.004), sex (*P* = 0.09; *R*^2^ = 0.05), or any moderator effect (*P* = 0.90; *R*^2^ = 0.05). For peak $\text{P}{\text{ET}}_{\text{C}{\text{O}}_{2}}$, there was no increase in explained variance with the addition of physical activity (*R*^2^ = 0.07; *P* = 0.05).

ICA dilation was not explained by the magnitude of the SR_AUC_ (*P* = 0.60; *R*^2^ = −0.005) or SR_AUC-initial_ (*P* = 0.25; *R*^2^ = 0.02); therefore, ICA dilation (%) was not normalized to shear rate. This held true at all age groups when analyses were investigated in young (*P* > 0.11), middle (*P* > 0.69), and older (*P* > 0.23) groups separately for SR_AUC_ and SR_AUC-initial_.

Hypercapnic peak responses for the ICA are shown in Table 3. For percent dilation of the ICA (allometrically scaled), there was no relationship with age (*P* = 0.07; Fig. 3*B*). With the addition of sex to the model, there was no relationship (*P* = 0.56), and no significant moderator effect was observed for ICA dilation (%; *P* = 0.24).

Mean ICA blood flow and the percent change in mean blood flow all showed a positive relationship with age (*P* ≤ 0.03). However, no sex (*P* ≥ 0.20) or moderator effects were observed (*P* ≥ 0.10).

The percent change in peak systolic ICA velocity showed no relationship with age (*P* = 0.21). With the addition of sex to the model, there was no relationship (*P* = 0.43); however, sex moderated the effect of age on percent change ICA velocity (*P* = 0.02). Simple slopes analyses revealed that there was a positive relationship between age and peak ICA velocity in males (β = 0.78 ± 0.29; *P* = 0.02) but not in female participants (β = −0.20 ± 0.29; *P* = 0.10).

**Figure 4.**
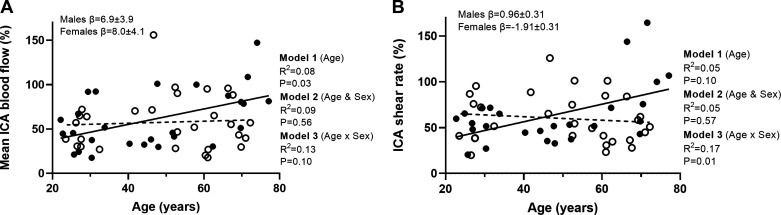
*A* and *B*: linear regression analysis demonstrating the relationships between age and ICA percent change in mean blood flow (*A*) and ICA percent change in shear rate (*n =* 58, females = 31; *B*). Solid line represents the regression fit for males, and dotted line represents the regression fit for female participants. Hierarchical regression models (*P* value and *R*^2^) are presented for the relationship with age (*model 1*), the relationship with sex (*model 2*), and the moderator relationship of the sex-dependent relationship with age (*model 3*). Slope coefficients (β) of the relationship with age are presented for males and females separately (*model 3*). ICA, internal carotid artery.

Peak shear rate showed no relationship with age (*P* = 0.97; *R* = −0.005); however, with the addition of sex to the model, this showed a relationship with shear rate higher in female participants and the model explaining 13% of variance (*P* = 0.008). No significant moderator effect of age was observed for peak shear rate (*P* = 0.11). As a percent change in shear rate, no relationship with age (*P* = 0.10) or sex (*P* = 0.57) was observed; however, sex was shown to moderate the relationship with age (*P* = 0.01). Simple slopes analyses revealed that there was a positive linear relationship in males with age (*P* = 0.003; β = 0.96 ± 0.31) and a negative relationship with age in females for shear rate as a percent change (*P* = 0.01; β = −1.91 ± 0.31; Fig. 4).

### Intracranial Kinetics Analyses

Dynamic onset response data are shown in Table 4. The MCAv response was well fitted by an exponential model (standard error of the τ: 2.46 ± 1.43). For all kinetic analysis outcomes of interest, there was no increase in explained variance with the addition of physical activity and BMI to the regression model (*R*^2^ < 0.023; *P* > 0.23) and therefore were not included in the full model.

**Table 4.** Intracranial responses to hypercapnia

	*Model 1*: Age			*Model 2*: Age and Sex					*Model 3*: Interaction (Age × Sex)			
				Age		Sex						
	*P* value	*R* ^2^	β	*P* value	β	*P* value	β	Combined *R*^2^	*P* value	*R* ^2^	β Males	β Females
*Kinetic parameters (n = 71, females = 38)*												
MCAv τ, s	**0.013**	0.09	0.28 ± 0.11	**0.023**	0.25 ± 0.11	0.13	5.79 ± 3.77	0.12	0.77	0.12	0.28 ± 0.15	0.22 ± 0.15
MCAv ΔAmp, cm/s	0.49	0.007	0.035 ± 0.05	0.72	0.018 ± 0.05	**0.03**	3.87 ± 1.74	0.08	0.31	0.09	−0.033 ± 0.071	0.07 ± 0.07
MCAv TD, s	0.05	0.05	0.095 ± 0.048	0.05	0.10 ± 0.049	0.52	−1.12 ± 1.71	0.06	0.13	0.09	0.18 ± 0.07	0.026 ± 0.07
MCAv MRT, s	**0.002**	0.13	0.37 ± 0.12	**0.004**	0.35 ± 0.12	0.26	4.67 ± 4.10	0.15	0.37	0.16	0.46 ± 0.17	0.25 ± 0.17
$\text{P}{\text{ET}}_{\text{C}{\text{O}}_{2}}$ τ, s	0.81	0.001	0.018 ± 0.08	0.79	0.021 ± 0.08	0.84	−0.54 ± 2.63	0.001	0.52	0.008	−0.03 ± 0.11	0.07 ± 0.11
$\text{P}{\text{ET}}_{\text{C}{\text{O}}_{2}}$ ΔAmp, mmHg	**0.004**	0.11	0.042 ± 0.01	**0.003**	0.044 ± 0.01	0.41	−0.42 ± 0.50	0.12	0.64	0.12	0.05 ± 0.02	0.04 ± 0.02
$\text{P}{\text{ET}}_{\text{C}{\text{O}}_{2}}$ TD, s	0.77	0.001	0.014 ± 0.05	0.81	0.012 ± 0.05	0.71	0.63 ± 1.69	0.003	0.13	0.04	0.086 ± 0.07	−0.062 ± 0.07
$\text{P}{\text{ET}}_{\text{C}{\text{O}}_{2}}$ MRT, s	0.69	0.002	0.033 ± 0.08	0.70	0.032 ± 0.05	0.97	−0.09 ± 2.80	0.002	0.76	0.004	0.06 ± 0.12	0.007 ± 0.12

Values are means ± SD. Boldface indicates significant relationship (*P* < 0.05). *Model 1* presents the relationship between age and the indicated variable. *Model 2* presents the addition of sex to the model and the relationship between age and sex with the indicated variable. *Model 3* indicates if sex moderates the relationship between age and the indicated variable, with individual β coefficients shown for males and females. In *models 1–3*, the *R*^2^ value reflects the full model. In *model 2*, the β coefficient provides the difference in females vs. males in the variable units. Amp, amplitude; MCAv, middle cerebral artery velocity; MRT, mean response time; $\text{P}{\text{ET}}_{\text{C}{\text{O}}_{2}}$, end-tidal carbon dioxide; τ, time constant; TD, time delay.

**Figure 5.**
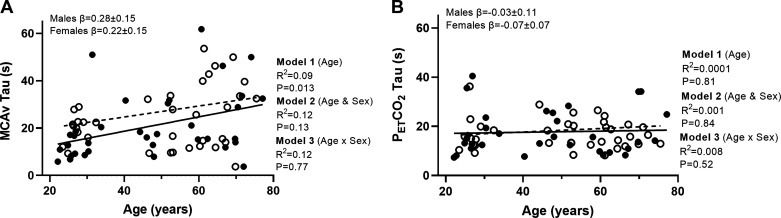
*A* and *B*: linear regression analysis demonstrating the relationships between age and MCAv time constant (τ; *A*) and $\text{P}{\text{ET}}_{\text{C}{\text{O}}_{2}}$ time constant (*n =* 71, females = 38; *B*). Solid line represents the regression fit for males, and dotted line represents the regression fit for female participants. Hierarchical regression models (*P* value and *R*^2^) are presented for the relationship with age (*model 1*), the relationship with sex (*model 2*), and the moderator relationship of the sex-dependent relationship with age (*model 3*). Slope coefficients (β) of the relationship with age are presented for males and females separately (*model 3*). MCAv, middle cerebral artery velocity; $\text{P}{\text{ET}}_{\text{C}{\text{O}}_{2}}$, end-tidal carbon dioxide.

For the MCA time constant, there was a positive relationship with age, such that the speed of the MCA response was slower with advancing age (*P* = 0.013). With the addition of sex to the model, this did not explain any variance in the response (*P* = 0.13), and no moderator effect of sex was observed for the MCAv time constant (*P* = 0.77; Table 4, Fig. 5*A*). For the $\text{P}{\text{ET}}_{\text{C}{\text{O}}_{2}}$ time constant, there was no relationship with age (*P* = 0.81; Fig. 5*B*). With the addition of sex to the model, no relationship was observed (*P* = 0.84) and no moderator effect (*P* = 0.52).

**Figure 6.**
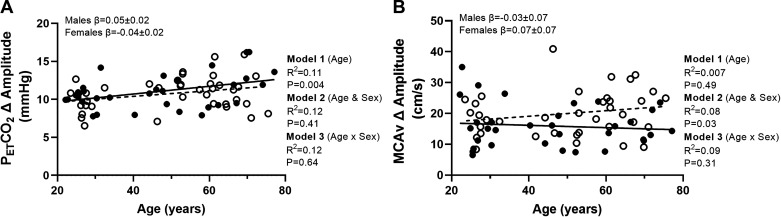
*A* and *B*: linear regression analysis demonstrating the relationships between age and $\text{P}{\text{ET}}_{\text{C}{\text{O}}_{2}}$ amplitude (*A*) and MCAv amplitude (*n =* 71, females = 38; *B*). Solid line represents the regression fit for males, and dotted line represents the regression fit for female participants (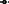). Hierarchical regression models (*P* value and *R*^2^) are presented for the relationship with age (*model 1*), the relationship with sex (*model 2*), and the moderator relationship of the sex-dependent relationship with age (*model 3*). Slope coefficients (β) of the relationship with age are presented for males and females separately (*model 3*). MCAv, middle cerebral artery velocity; $\text{P}{\text{ET}}_{\text{C}{\text{O}}_{2}}$, end-tidal carbon dioxide.

For the amplitude of the MCA response expressed as an absolute change, there was no relationship between the response and age (*P* = 0.49; Fig. 6*B*). With the addition of sex to the model, this showed a relationship with MCAv amplitude higher in female participants and the model explaining 8% the variance (*P* = 0.03). However, no moderator effect was observed (*P* = 0.31). The amplitude of the $\text{P}{\text{ET}}_{\text{C}{\text{O}}_{2}}$ response showed a positive relationship with age (*P* = 0.004). There was no relationship with sex (*P* = 0.41) and no moderator effect (*P* = 0.64; Fig. 6*A*).

For the MCAv MRT, there was a positive relationship with age, such that the speed of the response was slower with advancing age (*P* = 0.002). With the addition of sex to the model, this did not explain any additional variance (*P* = 0.26), and no moderator effect of sex was observed for the MCAv MRT (*P* = 0.37). For the $\text{P}{\text{ET}}_{\text{C}{\text{O}}_{2}}$ MRT, there was no relationship with age (*P* = 0.69). With the addition of sex to the model no relationship was observed (*P* = 0.97), and no moderator relationship was observed for the $\text{P}{\text{ET}}_{\text{C}{\text{O}}_{2}}$ MRT (*P* = 0.76).

As a pooled data set, there was a positive relationship between the MCAv and $\text{P}{\text{ET}}_{\text{C}{\text{O}}_{2}}$ time constants (*R*^2^ = 0.29; *P* = 0.013). When split into age categories, there was a positive relationship present in young adults (*R*^2^ = 0.62; *P* = 0.001); however, this relationship was lost in middle (*R*^2^ = 0.25; *P* = 0.18) and older aged adults (*R*^2^ = 0.13; *P* = 0.39).

### Relationships between Baseline and Hypercapnic Variables and Menopausal Status

The relationships between menopausal status and variables of interest are shown in Table 5. There was a negative relationship between MCAv and menopausal stage (*P* = 0.03; *R*^2^ = 0.18). Simple slopes showed the decrease in MCAv was different in late postmenopausal females compared with premenopausal females (*P* = 0.02); however, early postmenopausal females showed no difference in slope coefficients to premenopausal females (*P* = 0.78).

**Table 5.** Relationships between menopausal status and cerebrovascular variables

	Model		Coefficients			
	*P* value	*R* ^2^	Early postmenopausal		Late postmenopausal	
			β	*P* value	β	*P* value
*n*			7		15	
*Baseline intracranial parameters*						
MCAv, cm/s	**0.03**	0.18	2.0 ± 7.0	0.78	−13.0 ± 5.2	**0.02**
MAP, mmHg	**0.01**	0.22	5.2 ± 6.4	0.42	15.1 ± 4.7	**0.003**
CVCi, cm/s/mmHg	**0.002**	0.30	−0.05 ± 0.09	0.57	−0.24 ± 0.06	**< 0.001**
CVRi, mmHg/cm/s	**<0.001**	0.35	0.07 ± 0.19	0.73	0.59 ± 0.14	**< 0.001**
*Baseline cardiovascular parameters*						
$\text{P}{\text{ET}}_{\text{C}{\text{O}}_{2}}$, mmHg	0.72	0.018	−3.2 ± 4.2	0.45	−1.6 ± 3.1	0.60
PI, AU	**0.001**	0.31	−0.004 ± 0.05	0.94	0.13 ± 0.04	**0.001**
*Baseline extracranial internal carotid artery parameters*						
Diameter, cm	0.08	0.16	0.08 ± 0.05	0.09	0.05 ± 0.03	0.07
Shear rate, s^−1^	0.49	0.05	−29.4 ± 54.9	0.60	−39.6 ± 33.3	0.24
Mean blood flow, mL/min	0.21	0.11	289.6 ± 166.3	0.09	94.8 ± 103.5	0.37
Peak velocity, cm/s	0.83	0.013	4.2 ± 7.2	0.57	1.8 ± 4.8	0.70
*Peak intracranial parameters*						
MCAv, cm/s	0.11	0.12	−1.1 ± 9.6	0.89	−15.0 ± 7.1	**0.04**
MAP, mmHg	**0.009**	0.23	5.4 ± 6.7	0.43	16.4 ± 5.0	**0.002**
CVCi, cm/s/mmHg	**0.003**	0.28	−0.07 ± 0.10	0.48	−0.27 ± 0.07	**0.001**
CVRi, mmHg/cm/s	**0.005**	0.21	0.06 ± 0.17	0.73	0.43 ± 0.13	**0.002**
*Peak extracranial internal carotid artery parameters*						
ICA dilation, %	0.17	0.12	1.2 ± 1.8	0.51	2.2 ± 1.1	0.06
ICA mean blood flow, mL/min	0.16	0.13	447.8 ± 227.1	0.06	111.1 ± 141.4	0.79
ICA mean blood flow, %	0.71	0.03	12.5 ± 19.5	0.53	−4.3 ± 12.1	0.73
ICA shear rate, s^−1^	0.31	0.02	−22.2 ± 83.9	0.79	−79.6 ± 50.9	0.13
Peak systolic velocity, cm/s	0.59	0.04	9.5 ± 10.4	0.37	−1.4 ± 7.0	0.84
Peak systolic velocity, %	0.22	0.04	9.6 ± 15.2	0.53	−14.4 ± 10.2	0.17
SR, %	0.41	0.06	10.7 ± 17.8	0.55	−11.0 ± 10.8	0.32
SR _AUC_, AU	0.18	0.12	−11,279.7 ± 11,174.3	0.32	−10,353.1 ± 5,689.7	0.08
*Kinetic parameters*						
MCAv τ, s	0.78	0.014	0.74 ± 9.5	0.94	4.6 ± 6.6	0.12
MCA ΔAmp, cm/s	0.25	0.08	−3.7 ± 3.6	0.32	2.4 ± 2.5	0.34
$\text{P}{\text{ET}}_{\text{C}{\text{O}}_{2}}$ τ, s	0.99	0.001	0.8 ± 5.1	0.87	0.2 ± 3.8	0.96
$\text{P}{\text{ET}}_{\text{C}{\text{O}}_{2}}$ ΔAmp, mmHg	0.25	0.08	−3.7 ± 3.6	0.32	2.4 ± 2.5	0.34

Values are means ± SD. Boldface indicates significant relationship (*P* < 0.05). β Coefficients for early postmenopausal and late postmenopausal females are presented compared with reference group of premenopausal females. Amp, amplitude; CVR, cerebrovascular reactivity; CVRi, cerebrovascular resistance index; MAP, mean arterial pressure; MCAv, middle cerebral artery velocity; $\text{P}{\text{ET}}_{\text{C}{\text{O}}_{2}}$, end-tidal carbon dioxide; PI, pulsatility index; SR_AUC_, shear rate area under the curve; τ, time constant; TD, time delay; V̇e, minute ventilation.

## DISCUSSION

This is the first study to document the cross-sectional relationships of age and sex on kinetic responses to hypercapnia. The primary findings were that *1*) despite a negative relationship between age and absolute peak MCAv to hypercapnia, the relative amplitude-based responses to hypercapnia in the MCA and ICA reactivity were preserved with age; *2*) a positive relationship between age and the MCAv τ kinetic response, suggesting that age slows down the speed at which MCAv increases in response to a hypercapnic challenge; and *3*) reduced baseline and peak MCAv in late postmenopausal females, but no differences in ICA blood flow or CVR compared with premenopausal females.

### Resting Cerebral Blood Flow with Age

Declines in resting CBF with advancing age have been well documented (51–54). The current data corroborates this, documenting decreases in baseline MCAv with advancing age by ∼3 cm/s per decade alongside an increased CVRi. Despite a higher baseline MCAv in females than males, the relationship between age and MCAv was similar between males and females. This implies that the declines observed with aging were similar in both males and females with no age-by-sex interaction evident. Previous research has documented higher MCAv in young females; however, there was a greater rate of decline with advancing age (55, 56). This is contrary to the current study where we found preserved MCAv and CVRi in early postmenopausal females with 13 cm/s lower MCAv in late menopausal females; thus, declines in females in the current sample were driven by postmenopausal females. Differences between studies may be due to a younger average female sample in the current data with prior research comparing young versus older adults at extremes of the aging spectrum.

Declines in resting MCAv with advancing age may be due to numerous factors inclusive of decreased brain volume, cerebral metabolism, increases in arterial stiffness, oxidative stress, and decreased nitric oxide (NO) bioavailability (57–60). The greater declines in females are likely related to the loss of estrogen with the onset of menopause, which plays a pivotal role in mediating vascular function and blood pressure via the production of NO (61–63). However, the effects of the menopausal transition and loss of estrogen are less well documented in the cerebral vessels (64). Future research whereby blood markers for female sex hormones and NO concentrations are sampled is required to further understand the effects of menopause disentangled from the effects of age on the cerebrovascular circulation. In addition, in premenopausal females, large intra- and interindividual differences in estrogen concentrations prevail, even when controlling for cycle phase (65). Thus, direct measures of hormone concentrations in larger sample sizes are required in future investigations to account for this potential added variability in female responses and to fully understand the effects of female sex hormones on the cerebral circulation.

Despite observing a negative relationship between MCAv and age, baseline ICA mean blood flow showed no relationship with advancing age. This is in line with previous findings (66–68). It is proposed that declines in the posterior circulation with advancing age are more marked compared with the anterior circulation (68, 69). A potential explanation for the preserved mean ICA blood flow response may be due to the high physical activity levels in the current sample (∼2,182 METmin/wk), with regular physical activity shown to attenuate the age-related decline in CBF (70, 71) and endurance exercise training shown to increase CBF and CVR responses (72). When accounted for as a covariate, physical activity did not show a relationship with ICA mean flow; however, physical activity levels in the current sample were all relatively high across the sample (89% met or exceeded the PA guidelines), and therefore, comparisons on the influence of physical activity levels are limited and do not negate the potential for physical activity to influence the current results. Aerobic fitness levels were not measured in the current study, which may be a better predictor of both endothelial function and cognitive function (73, 74). In particular, a recent study has documented a positive relationship between fitness and resting MCAv in females but not in males; however, this relationship was no longer significant when adjusted for age (75). Further research investigating the relationships of age and sex on cerebral responses should therefore include direct measures of cardiorespiratory fitness to fully understand the effects of aging and sex on the cerebral circulation and any age- and sex-specific interactions with cardiorespiratory fitness.

### Cerebrovascular Reactivity and Age

Contrary to our hypotheses, MCAv CVR and %ICA reactivity showed no association with age. Interestingly, however, when included as a covariate, MAP showed a significant relationship with CVR. With the addition of MAP, this did not alter the relationships between CVR with age and sex and therefore indicates the effect of MAP is independent of the effects of age and sex. However, when blood pressure was factored into the hypercapnic response, presented as MCAv CVC, this showed a reduced response with age. It therefore seems that in older adults, there is a reliance on increased perfusion pressure to increase CBF to hypercapnia, consistent with recent data (6, 14, 28, 76). Despite similar absolute responses, current and previous data highlight regulatory differences with advancing age, inclusive of increased cerebrovascular resistance during the vasodilatory CO_2_ stimulus. Our findings are in line with Oudegeest-Sander et al. (9), Ito et al. (10), Murrell et al. (11), and Stefanidis et al. (12), who all observed preserved responses to hypercapnia with advancing age. However, research is conflicting with others reporting declines in CVR, in both the MCA and ICA (6, 20, 77). The study by Miller et al. (6) was able to account for MCA diameter changes and highlighted the ability of the MCA to dilate in young adults during hypercapnia, but not in older adults. This again indicates distinct regulatory differences with age. It suggests that using TCD-based measures of the MCA may underestimate flow and thus MCAv CVR in our younger adults, masking any potential alterations with age.

The current findings of no relationship between ICA reactivity and age are observed despite an increased mean blood flow and velocity in the ICA. Therefore, despite a potentially greater blood flow stimulus and perfusion pressure, no alterations in dilation of the ICA were observed with age. This may be indicative of an impaired response, with a greater stimulus required to elicit similar responses. Shear rate has been previously shown as the driving stimulus for changes in ICA dilation during hypercapnia (20). However, in the current study, there was no relationship between ICA dilation and shear stress, irrespective of age. This is in line with emerging evidence, highlighting that CVR assessed via steady-state CO_2_ does not reflect endothelial NO-dependent dilation (78). Given that this metric was not solely an endothelium-mediated measure of cerebrovascular function, this may explain why no alterations were found with advancing age.

Endothelial shear stress is an important regulator of vessel tone, mediating alterations in vessel structure, and function (79). In addition, declines in shear stress have been highlighted as an independent predictor of cognitive decline in older adults (80). In the present study, baseline shear rate was higher in females than males with no effect of age. We did observe a trend for a lower shear stress with older age; however, this was not significant. This is contrary to previous research that shows decreases in shear stress in cerebral arteries with age (81, 82). These decreases in shear rate with aging occur alongside increases in baseline diameter (82). However, the present study did not find increases in diameter with age and therefore may explain the preserved shear rate with age. This is in line with Iwamoto et al. (20) who observed no changes in baseline shear rate or diameter in older versus younger adults. It therefore seems that in the present sample population, vascular remodeling and increases in arterial diameter have not yet manifested, potentially because of the good health status and high physical activity levels reported. For shear rate responses to hypercapnia, no aging effect was seen, similar to previous findings (20). However, we did observe a sex-dependent effect of aging on shear rate responses, with an increase in males and a blunted response in females.

A notable difference with advancing age was the slowed MCAv speed of response (τ, MRT), observed in both male and female participants. These data indicate that despite a maintained capacity to obtain the same relative increase in MCAv, advancing age slows down the rate at which this response occurs. Reasons for this blunted MCAv τ and MRT with aging are largely speculative; however, it seems that in a healthy sample where cerebrovascular function remains intact, the time course of the response may reveal impairments in vasomotor control of the cerebrovasculature. Given the cerebrovaculature comprises numerous integrative mechanisms governing cerebrovascular control to ensure adequate CBF is maintained (83), this delayed response may be reflective of a compensatory response and reliance on differential mechanistic pathways to meet the demands of the brain. The MCAv τ was shown to be related to the $\text{P}{\text{ET}}_{\text{C}{\text{O}}_{2}}$ τ in young adults; however, this relationship was lost with advancing age. This highlights distinct regulatory differences with advancing age and indicates the blunted response with age was not due to the ventilatory response and may be due to other cerebral factors, inclusive of decreased cerebral metabolism, increased arterial stiffness, reduced compliance, slower autoregulatory responses, and greater reliance on perfusion pressure.

This study provides a comprehensive assessment of the age- and sex-dependent cerebrovascular responses to hypercapnia, using intra- and extracranial assessments in an adequately powered study design. Despite the novelties, the limitations of the current study should be acknowledged. First, TCD measures the velocity of the MCA and not absolute flow (4). Although the two are highly correlated (84) and prior work has shown the MCA diameter to remain constant during moderate elevations in MAP and $\text{P}{\text{ET}}_{\text{C}{\text{O}}_{2}}$ as seen in the current study (26, 85, 86), more recent evidence suggests this may not be the case (27, 87), particularly in the context of aging (6). Despite the draw backs of TCD, the higher temporal resolution provides possibilities for novel assessments of the dynamic responses to hypercapnia which the current study employs. Furthermore, the current study was unable to use prospective end-tidal targeting to standardize the end-tidal to arterial CO_2_ gradient (4). Therefore, the potential for differences in MCA and ICA reactivity between individuals may have been due to differing $\text{P}{\text{ET}}_{\text{C}{\text{O}}_{2}}$ stimuli and ventilatory responses. However, we found no differences in $\text{P}{\text{ET}}_{\text{C}{\text{O}}_{2}}$ concentrations with age or sex in the current sample and therefore believe that the lack of end-tidal targeting did not have a marked effect. Also, by not using end-tidal targeting, we were able to explore the kinetic relationships between $\text{P}{\text{ET}}_{\text{C}{\text{O}}_{2}}$ and MCAv responses with age. A final consideration of the current study is that we did not include measures of hypocapnia-induced CVR, which may have offered additional insights into the aging response (88). Future research should therefore employ both hypo- and hypercapnia-induced CVR to discern the effects of age and sex on CVR across the entire ventilatory range. However, given there is a lack of standardization of hypocapnia measures of CVR, this should first be addressed. Future research should also include measures of the posterior circulation, given the presence of regional differences in cerebrovascular regulation (23, 89).

In conclusion, this study provides insight into the relationships between age and sex on MCA and ICA reactivity to hypercapnia. In addition, this is the first study to investigate the kinetic responses of the MCA to hypercapnia with advancing age. Our findings demonstrated that despite similar MCAv CVR and ICA reactivity, dynamic responses of MCAv were significantly blunted with aging. These novel findings highlight the need for further investigation into the effects of age and sex on CVR responses. In particular, longitudinal study designs in larger sample sizes, with direct measures of sex hormones in females and cardiorespiratory fitness across the cohort. This will aid in accounting for any individual variability in the age-related responses and advance current understanding on the influence of sex hormones and menopause on cerebrovascular responses.

## SUPPLEMENTAL DATA

10.6084/m9.figshare.20422593 Supplemental Table S1: https://doi.org/10.6084/m9.figshare.20422593.

## GRANTS

J.L.K. is supported by a QUEX institute scholarship (University of Queensland and University of Exeter partnership).

## DISCLOSURES

No conflicts of interest, financial or otherwise, are declared by the authors.

## AUTHOR CONTRIBUTIONS

J.L.K., B.B., A.R.B., J.S.C., and T.G.B. conceived and designed research; J.L.K., S.L.R., and F.K.P. performed experiments; J.L.K. analyzed data; J.L.K. interpreted results of experiments; J.L.K. prepared figures; J.L.K. drafted manuscript; J.L.K., B.B., A.R.B., S.L.R., F.K.P., J.S.C., and T.G.B. edited and revised manuscript; J.L.K., B.B., A.R.B., S.L.R., F.K.P., J.S.C., and T.G.B. approved final version of manuscript.
